# Analysis of Interior-Point Paths

**DOI:** 10.6028/jres.111.013

**Published:** 2006-04-01

**Authors:** J. Stoer

**Affiliations:** Institut für Angewandte Mathematik und Statistik, Universität Würzburg, Germany

**Keywords:** infeasible interior-point paths, linear complementarity problems, linear programs, semidefinite linear complementarity problems, semidefinite linear programs

## Abstract

Infeasible-interior-point paths are the main tools in interior-point methods for solving many kinds of optimization problems. These paths are usually parametrized by a penalty-parameter *r* ↓ 0 and further parameters describing their off-centrality and infeasiblilty. Starting with an early result of C. Witzgall et al. [12] in linear programming, this paper gives an overview on results concerning the existence of these paths, their analyticity and the limiting behavior of their derivatives as *r* ↓ 0, and this also for degenerate problems in the areas of linear programming, linear complementarity problems, and semi-definte programming.

## 1. Introduction

This paper deals with the analysis of certain paths that are the main tool in sequential unconstrained optimization techniques (SUMT) and interior point methods. These were first studied systematically by Fiacco and McCormick in [3] as a means to solve constrained minimization problems of the form

$$\inf\left\{f\left(u\right)|g_{i}\left(u\right)\leq 0,i=1,2\ldots ,m\right\}.$$ (1)

In particular, they used Frisch’s logarithmic penalty function (see Frisch [4], [5]) and studied paths *u*(*r*), *r* > 0, of local minimizers of the penalized problem

$$\inf\left\{f\left(u\right)-r\sum_{i}\log\left(-g_{i}\left(u\right)\right)|\;g_{i}\left(u\right)<0,\;i=1,2,\ldots ,m\right\}$$

as *r* ↓ 0. They investigated the local existence and uniqueness of these paths, their differentiability proper ties for *r* > 0 and the convergence of both *u*(*r*) and *u*′(*r*) for *r* ↓ 0, if *r* > 0 is small and *u*(*r*) is close to a local optimal solution u^*^ of problem (1). They used the crucial assumption that u^*^ satisfies the standard regularity conditions, as the existence of Lagrange multipliers, the first order necessary and the second order sufficiency condition for a local minimum, and the strict complementarity condition. These assumptions are natural and they allow the use of the implicit function theorem to analyze the path. However they also exclude degenerate problems of practical importance: These occur e.g. already with convex and even linear or quadratic problems (1) that have more than one optimal solution u^*^ or which, together with their optimal Lagrange multipliers, violate strict complementarity.

Another limitation of paths like *u*(*r*) is that they exist only for strictly feasible optimization problems (1). More general are *infeasible-interior-point paths* that use strictly feasible solutions of a family of suitably perturbed optimization problems that shrink to the unperturbed problem (1) as the path parameter *r* tends to zero. Again this poses the question of the existence of such paths and their limiting behavior, also for degenerate unperturbed problems.

It is the purpose of this paper to give an overview over some results that are relevant in this context.

In a joint paper [12] with P. T. Boggs and P. D. Domich, C. Witzgall was one of the first to deal also with degenerate problems in the context of linear programs. Their pioneering results will be outlined in Sec. 2. Similar and even sharper results have been found for much more general classes of programs. We describe some of them that refer to linear complementarity problems in Sec. 3. Still more difficult are semidefinite linear programs and semidefinite linear complementarity problems. Results for them are described in the final Sec. 4.

## 2. Linear Programs

Consider the dual linear programs

$$\begin{array}{ll} \left(P\right)\;\min\;c^{T}x & \left(D\right)\;\max\;b^{T}u\\ \;x:\;Ax=b & \;y,u:\;A^{T}u+y=c,\\ \;x\geq 0, & \;y\geq 0, \end{array}$$

where *A* = (*a*_1_, …, *a_n_*) is a real *m* × *n*-matrix of rank *m* with columns *a_i_*. Let *S_P_* and *S_D_* be the *optimal solution sets* of (*P*) and (*D*).

Consider for (*D*) the Frisch logarithmic penalty function and its minimum *u*(*r*) for *r* > 0,

$$u\left(r\right):=\arg\;\underset{u}{\min}\left\{-b^{T}u-r\sum_{i=1}^{n}\log\left(c_{i}-a_{i}^{T}u\right)|A^{T}u<c\right\}.$$

The path {*u*(*r*)}*_r_*_>0_, if it exists, is called the *dual central path*; then *u* = *u*(*r*) solves the equation

$$b=r\sum_{i=1}^{n}\frac{a_{i}}{c_{i}-a_{i}^{T}u}$$ (2)

A central result of [12] adapted to (*P*) and (*D*) is

**Theorem 1** Assume

- rank *A* = *m*,
- (*D*) *is strictly feasible*,
- *S_D_ is nonempty and bounded*.

*Then the path u*(*r*) *is well-defined for r* > 0 *and the following limits exist*:

$$u*:=\underset{r↓0}{\lim\;}u\left(r\right)\in S_{D},\;u^{\prime }\left(0\right):=\underset{r↓0}{\lim}\frac{u\left(r\right)-u*}{r}.$$

The limit point u^*^ is the analytic center of S_D_, and the function u(*r*) *is analytic in r for r* > 0.

Let

$$y\left(r\right):=c-A^{T}u\left(r\right),\;x_{i}\left(r\right):=\frac{r}{y_{i}\left(r\right)},\;i=1,\ldots ,n.$$

Then (*x*, *y*, *u*)(*r*), *r* > 0, the *primal-dual central path*, solves

$${\left(PD\right)}_{r}\;\begin{array}{l} \;Ax=b,\;x>0,\\ \;A^{T}u+y=c,\;y>0,\\ \;x\circ y=re, \end{array}$$

where *e* is the vector

$$e:={\left(1,1,\ldots ,1\right)}^{T}\in R^{n},$$

and *x* ○ *y* is the componentwise product

$$x\circ y:={\left(x_{1}y_{1},\cdots ,x_{n}y_{n}\right)}^{T}.$$

By Eq. 2 the primal-dual path exists in the situation of Theorem 1. In general, the primal-dual path is well-defined for *r* > 0, provided (*P*) and (*D*) are *strictly feasible*; primal-dual interior point methods try to follow this path for *r* ↓ 0. But there are some difficulties with this concept:

– (*PD*)*_r_* is a nonlinear equation for each *r*, so one can at best approximate points on it.
– The path does not exist, if (*P*) and (*D*) are not strictly feasible, even when they are solvable.

These difficulties are avoided if one uses *infeasible (weighted) interior-point paths* instead, defined as the solution *z*: = (*x*, *y*, *u*) = *z*(*r*, *η*) = (*x*, *y*, *u*)(*r*, *η*) of the nonlinear system

$${\left(PD\right)}_{r,\eta }\;\begin{array}{l} \;Ax=b+r\bar{b},\;x>0\\ \;A^{T}u+y=c+r\bar{c},\;y>0,\\ \;x\circ y=r\eta , \end{array}$$

where *r* > 0, 0 < *η* ∈ ***R****^n^* is a weight vector, and $\bar{b}$, $\bar{c}$ are suitable perturbation vectors. The infeasible interior-point *central path* belongs to the weight vector *e*.

As initialization one may choose *r*_0_: = 1, any *x*_0_ > 0, *y*_0_ > 0, *u*_0_, and put

$$\bar{b}:=Ax_{0}-b,\;\bar{c}:=A^{T}u_{0}+y_{0}-c,\;\eta_{0}:\;=x_{0}\circ y_{0}.$$

Then (*x*_0_, *y*_0_, *u*_0_) = (*x*, *y*, *u*)(*r*_0_, *η*_0_) solves at least (*PD*)*_r,η_* for *r* = *r*_0_ and *η* = *η*_0_. The choice *x*_0_ = *y*_0_: = *e* leads to *η*_0_ = *e*, that is to a point on the central path.

The practical significance of these paths lies in their use in *infeasible-interior-point methods* (see e.g. Mizuno [10] for an early analysis of such methods in the context of linear programming). Typically, these methods start with an initial point

$$z_{0}=z\left(r_{0},\eta_{0}\right),\;r_{0}>0,\;\eta_{0}>0,$$

on the initial trajectory at (*r*_0_, *η*_0_) defined by the initialization. Then these methods construct further iterates

$$z_{k}=z\left(r_{k},\eta_{k}\right),\;r_{k}>0,\;0<\eta_{k}\in R^{n},\;k=1,2,\ldots ,$$

where the *r_k_* > *r_k_*_+1_ > ⋯ > 0 converge to 0 and the weights *η_k_* ≈ *e* stay bounded and close to *e*. The construction of *z_k_*_+1_ from *z_k_* use, as a rule, the tangent of *z*(., *η_k_*) at *r_k_*, that is the derivative $z_{k}^{\prime }:=\partial z\left(r,\eta_{k}\right)/\partial r|{}_{r=r_{k}}$ (usually called the *affine scaling direction*). The quality of these methods depends on how quickly the *r_k_* converge to 0. With most methods, the superlinear convergence of the *r_k_* relies on the existence (and boundedness) of the derivatives *z_k_*′ = *z*′ (*r_k_*, *η_k_*) as *r_k_* ↓ 0.

Also with the paths *z*(*r*, *η*) the same questions arise as before:

– their existence and uniqueness for 0 < *r* ≤ *r*_0_ = 1, *η* > 0,
– their analytical properties,
– and their limiting properties for *r* ↓ 0.

We will answer these questions in the context of more general complementarity problems in Sec. 3. Here, we only formulate linear programs as particular complementarity problems, which is well-known.

By the duality theorem of linear programming, feasible solutions *x*, (*y*, *u*) of (*P*) and (*D*) satisfy

$$0\leq c^{T}x-b^{T}u={\left(A^{T}u+y\right)}^{T}x-{\left(Ax\right)}^{T}u=y^{T}x,$$

and they are optimal solutions of (*P*) and (*D*), if they are feasible and *x^T^y* = 0.

By elimination of *u*, define the linear manifold ℳ ⊂ ***R***^2^*^n^*,

$$ℳ:=\left\{\left(x,y\right)|\exists u:Ax=b,A^{T}u+y=c\right\},$$

and the associated linear subspace

$$\Phi :=\left\{\left(x,y\right)|\exists u:Ax=0,A^{T}u+y=0\right\}.$$

Then

$$(x,y)\in \Phi \Rightarrow x^{T}y=0,$$

and by the assumption rank (*A*) = *m*,

dimΦ=dimℳ=n.

The linear programs (*P*) and (*D*) are then equivalent to the *associated complementarity problem* of finding a solution (*x*, *y*) of

$$\left(LCP_{LP}\right)\;\begin{array}{c} \left(x,y\right)\in ℳ\\ x\geq 0,y\geq 0,\\ x\circ y=0\left(\Leftrightarrow x^{T}y=0\right). \end{array}$$

Under the assumptions of Theorem 1, this particular complementarity problem satisfies

$$\begin{array}{l} \left(a\right)\;\dim\Phi =\eta \\ \left(b\right)\;x^{T}y=0\;\text{for}\;\text{all}\;\left(x,y\right)\in \Phi \\ \left(c\right)\;\left(LCP_{LP}\right)\;\text{is}\;\text{solvable}. \end{array}$$ (3)

## 3. General Linear Complementarity Problems

A general (LCP) (in horizontal form) is to solve

$$\left(LCP\right)\;\begin{array}{c} Px+Qy=q,\\ x\geq 0,y\geq 0,\\ x\circ y=\left(\Leftrightarrow x^{T}y=0\right), \end{array}$$

where *q* ∈ ***R****^n^* and *P*, *Q* are real *n* × *n*-matrices with rank [*P*,*Q*] = *n*. With respect to (*LCP*) we define:

1. *z* = (*x*, *y*) is a (strictly) *feasible point* of (*LCP*), iff *Px* + *Qy* = *q* and *z* ≥ 0 (resp. *z* > 0).
2. The *set of solutions* of (*LCP*) is denoted by *S*. A solution (*x*, *y*) ∈ *S* is called *strictly complementary*, if *x* + *y* > 0.
3. Φ ⊂ ***R***^2^*^n^* is the linear space
  Φ:=N[P,Q]={(x,y)|Px+Qy=0}.
4. (*LCP*), resp. Φ, is called *monotone*, if
  $$\left(x,y\right)\in \Phi \Rightarrow x^{T}y\geq 0.$$

The *LCP*s associated with linear programs are monotone; by Eq. 3 they even satisfy *x^T^y* = 0 for all (*x*, *y*) ∈ Φ.

For the presentation of the following results, we make the following rather weak standard assumption

$$\left(V\right)\;\begin{array}{l} \;1)\;\text{rank}\left[P,Q\right]=n,i.e.\dim\Phi =n,\\ \;2)\;\left(\text{LCP}\right)\;\text{is}\;\text{monotone}\\ \;3)\;S\neq \emptyset ,\left(LCP\right)\;\text{is}\;\text{solvable}. \end{array}$$

Note that (*V*) 3) does not require the unique solvability of (*LCP*). Degenerate problems with more than one solution are not excluded.

Still more general than monotone complementarity problems are *sufficient* linear complementarity problems. They were introduced by Cottle, Pang, and Venkateswaran in [2] and further characterized by Väliaho [11]. They are the most general class of linear complementarity problems of the form (*LCP*) with the following properties:

1. For all *q*, the solution set *S* = *S*(*q*) of (*LCP*) is a convex (perhaps empty) set.
2. Each stationary solution of the (nonconvex) quadratic optimization problem
  $$\begin{array}{l} \;\text{inf}\left\{x^{T}y|\left(x,y\right)\;\text{is}\;a\;\text{feasible}\;\text{point}\;\text{of}\;\left(LCP\right)\right\}\\ \text{associated}\;\text{with}\;\left(LCP\right)\;\text{is}\;a\;\text{solution}\;\text{of}\;\left(LCP\right). \end{array}$$

Again, infeasible (weighted) interior-point paths play an important role in methods for solving (*LCP*). They are defined as the solutions (*x*, *y*) = (*x*, *y*)(*r*, *η*) of

$${\left(LCP\right)}_{r,\eta }\;\begin{array}{l} \;Px+Qy=q+r\bar{q},\left(x,y\right)>0,\\ \;x\circ y=r\eta , \end{array}$$

where *r* > 0, 0 < *η* ∈ *R^n^* is a weight, and $\bar{q}\;\in \;R^{n}$ is a perturbation vector.

For initialization, choose any *x*_0_ > 0, *y*_0_ > 0, *r*_0_ > 0, and put

$$\bar{q}:=\left(Px_{0}+Qy_{0}-q\right)/r_{0},\eta_{0}:=x_{0}\circ y_{0}/r_{0}.$$

Then at least (*x*_0_, *y*_0_) = (*x*, *y*)(*r*_0_, *η*_0_), and

$$\bar{q}\in M_{r_{0}}:=\left\{\left(Px+Qy-q\right)/r_{0}|x>0,y>0\right\}.$$

Interior-point methods for solving (*LCP*) try to follow such paths for *r* ↓ 0 in much the same way as described for linear programs in Sec. 2.

As before, the same questions with respect to these paths (*x*, *y*)(*r*, *η*) arise,

– their existence and uniqueness for 0 < *r* ≤ *r*_0_ = 1, *η* > 0,
– their analytical properties as functions of *r*, *η*,
– and their limiting properties for *r* ↓ 0.

The following theorem (see Stoer and Wechs [9]) can be viewed as a generalization of Theorem 1, the result of Witzgall et al.:

**Theorem 2** *Assume* (*V*), *r*_0_ > 0, *and* $\bar{q}\in M_{r_{0}}$. *Then the following holds*:

- *The path z*(*r, η*): = (*x, y*)(*r, η*) *is a well-defined analytic function of* (*r, η*) *for all* $\left(r,\eta \right)\in \left(0,r_{0}\right]\times R_{++}^{n}$, *which converges for r* ↓ 0 *to a solution of* (*LCP*):
  $$\exists z^{*}\left(\eta \right)=(x^{*},y^{*})\left(\eta \right):=\underset{r↓0}{\text{lim}}\left(x,y\right)\left(r,\eta \right)\in S.$$
- *If, in addition*, (*LCP*) *has a strictly complementary solution, then there is an ε* > 0, *so that the function z*(*r, η*) *can be extended to an analytic function* $\hat{z}(r,\eta )$ *on the enlarged domain of all* $\left(r,\eta \right)\in \left[–\varepsilon ,\;r_{0}\right]\times R_{++}^{n}$
- *If* (*LCP*) *is solvable but has no strictly complementary solution, then there is an ε* > 0, *and an analytic function* $\hat{z}\left(\rho ,\eta \right)$ *on the domain* $\left(\rho ,\eta \right)\in \left[-\varepsilon ,\sqrt{r_{0}}\right]\times R_{++}^{n}$ *so that* $z\left(\rho^{2},\eta \right)=\hat{z}\left(\rho ,\eta \right)$ *for all*
  $$\left(\rho ,\eta \right)\in (0,\sqrt{r_{0}}]\times R_{++}^{n}.$$
  (“(*x*, *y*)(*r*, *η*) is an analytic function of $\rho =\sqrt{r}$ even in *r* = 0.”)

### 3.1 Remarks

1. By a result of Goldman and Tucker [6], any solvable complementarity problem associated with linear programs has also a strictly complementary solution. So, part b) of the theorem applies in this case, which generalizes
  Theorem 1: the limits
  $$z^{*\left(k\right)}\left(\eta \right):=\underset{r↓0}{\lim}\frac{\partial^{k}}{\partial r^{k}}z\left(r,\eta \right),\;k=0,1,\ldots ,$$
  of all derivatives exist, and are in fact the derivatives of an analytic function $\hat{z}\left(r,\eta \right)$ at *r* = 0 extending *z*(*r*, *η*).
2. It is easy to find convex quadratic programs for which the corresponding complementarity problem is only solvable but has no strictly complementary solution, so case c) of the theorem can occur.
3. Theorem 2 remains true for sufficient complementarity problems (i.e. if “monotone” is replace by “sufficient” in Assumption (*V*) (see [8]).

The proof of Theorem 2 is not easy in the case of degeneracies, when (*LCP*) has more than one solution or no strictly complementary solution. The mathematical reason is that the solutions (*x*, *y*, *r*, *η*) of (*LCP*)*_r,η_* with *x* ≥ 0, *y* ≥ 0, *r* ≥ 0 and *η* > 0 then form a semialgebraic variety with a singularity in *r* = 0, so that the implicit function theorem cannot be applied directly (though indirectly, see [9], [8] for details).

## 4. Semidefinite Linear Programs and Complementarity Problems

In this section we show that the results of Theorems 1 and 2 even generalize to semidefinite linear programs and to semidefinite linear complementarity problems.

We use the following notation, which is standard:

*S^n^*: = {*A* ∈ ***R****^n^*^×^*^n^* | *A* = *A^T^*} is the space of all real symmetric *n* × *n*-matrices.
*S^n^* is provided with the scalar product 〈*A*,*B*〉: = tr (*B^T^A*) = tr (*BA*).
*A* ≥ 0 (resp. *A* ≻ 0) means that *A* ∈ *S^n^* is positive semidefinite (resp. positive definite), and $S_{++}^{n}$ is the set of all positive definite matrices in *S^n^*.

Then the standard dual pair of semidefinite linear programs has the form

$$\left(P\right)\;\begin{array}{l} \;\inf\;\langle C,X\rangle \\ \;X:\langle A_{i},X\rangle =b_{i},i=1,2,\;\ldots ,m,\\ \;X≽0. \end{array}$$

$$\left(D\right)\;\begin{array}{l} \;\sup\;b^{T}u\\ \;u,Y:\sum_{i=1}^{m}u_{i}A_{i}+Y\\ \;Y≽0. \end{array}=C,$$

Here,

$$C,A_{i}\in S^{n}\;\text{for}\;i=1,\ldots ,m,\;b{\left(b_{1},\ldots ,b_{m}\right)}^{T}\in R^{m}.$$

The following results are known from the duality theory of semidefinite linear programs:

If (*P*) and (*D*) have strictly feasible solutions then they also have optimal solutions, and *X* and (*u*, *Y*) are optimal for (*P*) and (*D*), respectively, iff

$$\begin{array}{l} \;\langle A_{i},X\rangle =b_{i},i=1,2,\;\ldots ,m,\\ \;\sum_{i=1}^{m}u_{i}A_{i}+Y=C,\\ \;XY=0,X≽0,Y≽0. \end{array}$$

By elimination of *u* one can formulate this as a particular semidefinite linear complementarity problem, which in general has the following form: Solve

$$\begin{array}{l} \left(SDLCP\right)\;P\left(X\right)+Q\left(Y\right)=q,\\ \;XY=0,X,Y≽0. \end{array}$$

Here, $q\in R^{\bar{n}},\bar{n}:=n\left(n+1\right)/2=\text{dim}S^{n}$, and $P,Q:S^{n}\to R^{\bar{n}}$ are linear operators of the form

$$\begin{array}{l} P\left(X\right)={\left(\langle P_{1},X\rangle ,\;\ldots ,\langle P_{\bar{n}},X\rangle \right)}^{T},\;P_{i}\in S^{n},\\ Q\left(X\right)={\left(\langle Q_{1},X\rangle ,\;\ldots ,\langle Q_{\bar{n}},X\rangle \right)}^{T},\;Q_{i}\in S^{n}. \end{array}$$

As in the previous section we define:

1. The nullspace N([P,Q]) of [*P*,*Q*] is denoted by
  $$\Phi :=\left\{\left(X,Y\right)|P\left(X\right)+Q\left(Y\right)=0,X,Y\in S^{n}\right\}.$$
2. (*SDLCP*), resp. Φ, is called *monotone*, if
  (X,Y)∈Φ⇒〈X,Y〉≥0.
3. The set ℱ of *feasible points* of (*SDLCP*) is defined by
  ℱ:={(X,Y)|P(X)+Q(Y)=q,X,Y≽0}.
4. The set of *solutions* of *SDLCP* is denoted by
  S:={(X,Y)∈ℱ|XY=0}.
5. A solution (*X*, *Y*) ∈ *S* is called *strictly complementary* if *X* + *Y* ≻ 0.

In the case of the dual semidefinite linear programs (*P*), (*D*), Φ is given by

$$\Phi =\left\{\left(X,Y\right)|\langle A_{i},X\rangle =0,\;\text{for}\;i=1,\;\ldots ,m,Y=-\sum_{i=1}^{m}u_{i}A_{i}\;\text{for}\;\text{some}\;u\right\},$$

and Φ even satisfies

(X,Y)∈Φ⇒〈X,Y〉=0,

so the corresponding (*SDLCP*) is monotone. It is not known whether in this case the corresponding (*SDLCP*) always has a strictly complementary solution if it is solvable.

The following standard hypothesis, which corresponds to the standard assumption of the previous section, is very weak

$$\left(V\right)\;\begin{array}{l} \;1)\;\left\{P\left(X\right)+Q\left(Y\right)|X,Y\in S^{n}\right\}=R^{\bar{n}},\\ \;2)\;\left(SDLCP\right)\;\text{is}\;\text{montone}\\ \;3)\;S\neq \emptyset ,\;\left(SDLCP\right)\;\text{is}\;\text{solvable}. \end{array}$$

The definition of infeasible weighted interior-point paths differs slightly from the previous section: For 0 < *r* ≤ *r*_0_ and weight matrices *M* ≻ 0, infeasible weighted interior point paths are now defined as solutions (*X*, *Y*) = (*X*, *Y*)(*r*, *M*) of

$$\begin{array}{l} P\left(X\right)+Q\left(Y\right)=q+r\bar{q},\;X≻0,\;Y≻0\\ \left(XY+YX\right)/2=rM. \end{array}$$

Here, the condition *XY* = 0 of (*SDLCP*) is weakened to (*XY* + *YX*)/2 = *rM*, which corresponds to the “AHO” approach proposed by Alizadeh, Haeberly and Overton in [1].

The *perturbation vector* $\bar{q}$ is chosen such that $\bar{q}\in {ℳ}_{r_{0}}$ for some *r*_0_ > 0, where

$${ℳ}_{r}:=\left\{\frac{P\left(X\right)+Q\left(Y\right)-q}{r}|X,Y,XY+YX≻0\right\}.$$

A possible initialization is to choose *r*_0_: = 1, *X*_0_ = *Y*_0_: = *I* and *M*_0_: = *I* and $\bar{q}:=P\left(X_{0}\right)+Q\left(Y_{0}\right)–q$.

Again the same questions arise for these paths (*X*, *Y*)(*r*, *M*) as in the previous sections, namely their

– existence and uniqueness for 0 < *r* ≤ *r*_0_, *M* ≻ 0,
– analytical properties as functions of *r* and *M*,
– and their limiting properties for *r* ↓ 0.

The following is the main result on (*SDLCP*). It corresponds to Theorems 1 and 2, and shows the fruitfulness of the early results of C. Witzgall:

**Theorem 3** *Assume* (*V*), *r*_0_ > 0 *and* $\bar{q}\in {ℳ}_{r_{0}}$. *Then*:

1. *The path function* (*X,Y*)(*r, M*) *is a well-defined analytic function for all* $\left(r,M\right)\in \left(0,\;r_{0}\right]\times S_{++}^{{}^{n}}$, *which converges to a solution of* (*SDLCP*) *as r* ↓ 0,
  $$\exists \left(X^{*},Y^{*}\right)\left(M\right):=\underset{r↓0}{\lim}\left(X,Y\right)\left(r,M\right)\in S.$$
2. *If* (*SDLCP*) *even has a* strictly complementary solution, *then there is an ε* > 0 *so that the path function* (*X, Y*)(*r, M*) *can be extended to an analytic function* $\left(\hat{X},\hat{Y}\right)\left(r,M\right)$ *defined on the* enlarged domain $\left(r,M\right)\in \left(–\varepsilon ,\;r_{0}\right]\times S_{++}^{n}$.
  “(*X*, *Y*)(*r*, *M*) is analytic in (*r*, *M*) even at *r* = 0.”

The proof of this theorem is even more involved than that of Theorem 2, it can be found in Preiss and Stoer [7].

It is an open problem, whether (and how) the path function (*X*, *Y*)(*r*, *M*) can be extended, if (*SDLCP*) satisfies only (*V*), but has no strictly complementary solutions.
